# Screening of Agelasine D and Analogs for Inhibitory Activity against Pathogenic Protozoa; Identification of Hits for Visceral Leishmaniasis and Chagas Disease

**DOI:** 10.3390/molecules14010279

**Published:** 2009-01-08

**Authors:** Anders Vik, Ágnes Proszenyák, Marieke Vermeersch, Paul Cos, Louis Maes, Lise-Lotte Gundersen

**Affiliations:** 1 Department of Chemistry, University of Oslo, P.O.Box 1033, Blindern, N-0315 Oslo, Norway; E-mails: vik.anders@gmail.com (A. V.), agnes.proszenyak@kjemi.uio.no (A. P.); 2 University of Antwerp, Laboratory of Microbiology, Parasitology and Hygiene, Faculty of Pharmaceutical, Biomedical and Veterinary Sciences, Universiteitsplein 1, B-2610 Antwerp, Belgium; E-mails: marieke.vermeersch@ua.ac.be (M. V.), paul.cos@ua.ac.be (P. C.), louis.maes@ua.ac.be (L. M.); 3 Institute for Tropical Medicine, Nationalestraat 155, B-2000 Antwerp, Belgium

**Keywords:** Agelasine, Antiprotozoal, Chagas disease, Visceral leishmaniasis.

## Abstract

There is an urgent need for novel and improved drugs against several tropical diseases caused by protozoa. The marine sponge (*Agelas* sp.) metabolite agelasine D, as well as other agelasine analogs and related structures were screened for inhibitory activity against *Plasmodium falciparum, Leishmania infantum, Trypanosoma brucei* and *T. cruzi*, as well as for toxicity against MRC-5 fibroblast cells. Many compounds displayed high general toxicity towards both the protozoa and MRC-5 cells. However, two compounds exhibited more selective inhibitory activity against *L. infantum* (IC_50_ <0.5 μg/mL) while two others displayed IC_50_ <1 μg/mL against *T. cruzi* in combination with relatively low toxicity against MRC-5 cells. According to criteria set up by the WHO Special Programme for Research & Training in Tropical Diseases (TDR), these compounds could be classified as hits for leishmaniasis and for Chagas disease, respectively*.* Identification of the hits as well as other SAR data from this initial screening will be valuable for design of more potent and selective potential drugs against these neglected tropical diseases.

## Introduction

Several so-called neglected diseases, *e.g*. illnesses that disproportionally affect poor and marginalized populations and for which satisfactory treatment is not available, partly due to lack of interest in drug development, are due to protozoal infections. Major killers in developing countries include malaria [1,2], visceral leishmaniasis or kala-azar [3,4], African sleeping sickness [4,5] and American sleeping sickness or Chagas disease [6]. Several factors limit the utility of existing drugs in areas were they are really needed, for instance high cost, poor compliance, drug resistance, low efficacy and toxicity [7]. Current drugs used to treat *Trypanosoma* infections are unsatisfactory with respect to safety [6] and an increasing number of malaria cases are caused by *Plasmodium falciparum* resistant to first-line drugs [1,2]. Hence, there is a constant need for development of novel antiprotozoal drugs, and one strategy in search for new hits and leads is screening of natural products, including those found in marine organisms [8].

**Figure 1:**
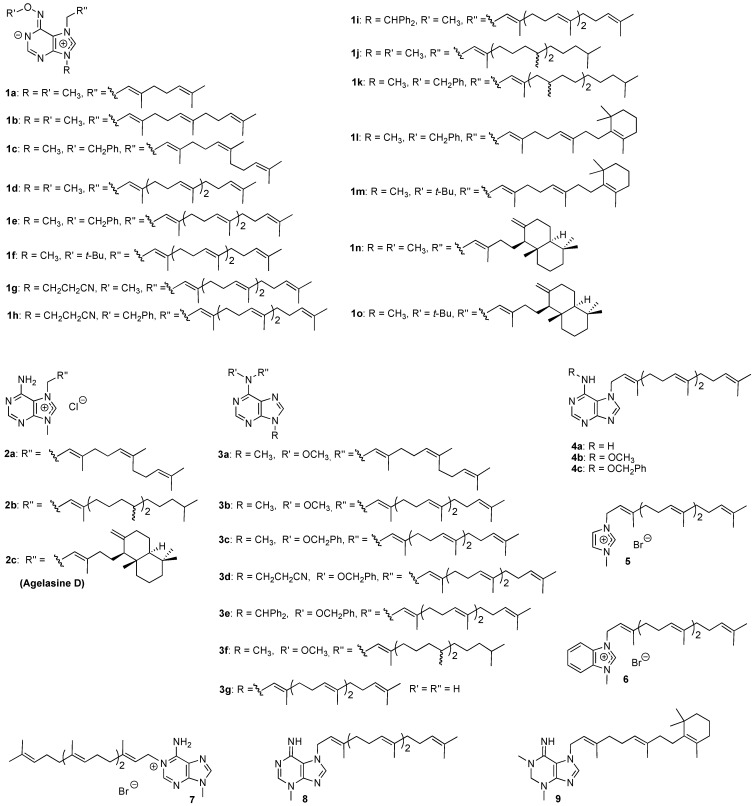
Structures of the studied agelasine analogs **1**-**9**.

Agelasines are isolated from marine sponges (*Agelas* sp) [9,10,11,12,13,14,15,16,17]. We have completed the first syntheses of agelasine D [18,19], agelasine E [20] and *ent*-agelasine F [21] as well as several synthetic analogs [18,19,20,22,23,24,20,22]. It has been shown that many of these compounds possess a broad spectrum of biological activities; including cytotoxicity towards cancer cell lines [19,22,23], antibacterial- [19,20,21,22,23], antifungal [23] and antifouling activities [25]. Recently, we also demonstrated that some agelasine analogs displayed antiprotozoal activity (*Acanthamoeba castellanii* and *A. polyphaga*) [22]. Hence we chose to screen agelasine D and some agelasine analogs and structurally related compounds for activity against pathogenic protozoa causing the above mentioned tropical neglected diseases. The structures of the compounds studied are shown in Figure 1. Compounds **8** and **9** may be regarded as analogs of agelasimines, another class of purine-containing natural products isolated from *Agelas* sp. [26,27].

## Results and Discussion

### Antiprotozoal activities

The marine sponge metabolite agelasine D (**2c**) was screened for *in vitro* activity against the pathogenic protozoa *P. falciparum, Leishmania infantum, Trypanosoma cruzi,* and *Trypanosoma brucei* (Table 1). To assess selectivity of action, cytotoxicity against MRC-5 fibroblast cells was also evaluated. A higher activity was found for agelasine D (**2c**; IC_50_ 0.29 μg/mL = 0.63 μM) against *P. falciparum* than what was recently reported for agelasines J (IC_50_ 6.6 μM), K (IC_50_ 8.3 μM), and L (IC_50_ 18 μM) [17]. The selectivity index for antimalarial action [SI; IC_50_(MRC-5 fibroblast)/IC_50_(*P. falciparum*)] was 23, and agelasine D (**2c**) displayed significant inhibitory action also against the other parasites examined. These results encouraged us to examine the antiprotozoal activities of a number of agelasine analogs **1** – **9** in search for more potent and selective compounds. The results are presented in Table 1. Compounds with high cytotoxicity and related aspecificity across the different models were not titrated down to the exact IC_50_ as they may never become relevant hits for further follow-up.

For an antimalaria hit, the WHO Special Programme for Research & Training in Tropical Diseases (TDR) defines an activity criterion to be IC_50_ <0.2 μg/mL with SI >20 [28]. Only compound **1d**, with IC_50_ = 0.10 μg/mL and SI = 20, met this requirement. However, this compound also showed comparable levels of activity against the other parasite species, suggesting aspecific action. A few other analogues [**1a** – **1c**, **1f**, **1j**, **1m** – **1o**, in addition to **2c** (agelasine D)] displayed IC_50_ values against *P. falciparum* in the range 0.2 – 1.0 μg/mL, while compounds **3** – **9** were in general only marginally active.

Compound **1d** was found to be a potent *in vitro* inhibitor of *L. infantum* with an IC_50_ value of 0.093 μg/mL and SI = 22. This compound meets the definition of a hit according to TDR (IC_50_ <0.5 μg/mL and SI >20) [28]. Compound **5** also classifies as a hit. We have previously shown that compound **1d** displays a rather broad spectrum of antibacterial activities, incl. inhibition of *Mycobacterium tuberculosis* [19,23], whereas the imidazole **5** were reasonably active against *Staphyllococcus aureus*, but not against *Escherichia coli* and *M. tuberculosis* [23]. Compounds **1f** and **1j** appeared also as potent inhibitors of *L. infantum*, but unfortunately these compounds were equally toxic to MRC-5 cells. Compounds **3** – **4** and **6** – **9** were in general only weakly active against *L. infantum.*

Quite a few of the agelasine analogs inhibited *T. cruzi* growth, as they displayed IC_50_ values <1 μg/mL [28], but particularly compounds **1** also exhibited profound cytotoxicity towards MRC-5 cells, which is also the host cell in the *T. cruzi* model. Nevertheless, compounds **2a** and **5**, with SI >50, could be classified as hits according to the definition given above. Agelasine analog **2a** is of special interest, since this compound, in contrast to the imidazole **5**, displays low inhibitory activity against the other protozoa. In addition, previous studies have revealed the compound **2a** is virtually inactive against bacteria (*S. aureus*, *E. coli* and *M. tuberculosis*) [23]. Moderate toxicity (SI = 10 – 30) and IC_50_ against *T. cruzi* <1 μg/mL were found also for compounds **1d**, **3f** and **9**.

None of the compounds examined qualified as a hit with respect to *T. b. brucei* (IC_50_ <0.2 μg/mL and SI >100) [28]. The majority of active compounds were far too toxic. The most interesting results were found for compound **5** with IC_50_ <0.11 μg/mL and SI >69.

### Structure – activity relationships

From the results in Table 1, it can be seen that type **1** compounds (Figure 1) generally exhibit a broad spectrum of antiprotozoal activities, but in an aspecific manner, since many of these compounds are equally toxic towards MRC-5 cells. Compounds with a monoterpene- (eg. **1a**), or sesquiterpene (**1b** and **1c**) derived side-chain in the purine 7-position appear to be more toxic, compared to some of the compounds with longer side-chains. Among the diterpenoids, a phytyl substituent (**1j** and **1k**) and the β-cyclocitral derived side-chain found in **1l** and **1m**, also results in aspecific activity. The most interesting compounds in this class have a geranylgeranyl side-chain or the same diterpenoid substituent as found in agelasine D (**2c**). Compounds **1d** and **1n**, both carrying a methoxy group at *N*^6^ and a methyl group at *N*-9, were identified as antileishmanial hits. A trend seems to be that changing the *N*^6^ substituents from methoxy to a benzyloxy- (compounds **1c**, **1e**, **1h**, **1k**, and **1l**) or *tert*-butoxy group (**1f**, **1m**, and **1o**) generally results in compounds with enhanced selectivity towards *Trypanosoma* sp., compared to the other parasites, but this modification does not result in significant reduction of MRC-5 cytotoxicity. Similar results were obtained when the *N*-9 substituent differed from methyl (**1g** – **1i**).

Agelasine D and analogs **2** have a primary amino group in the purine 6-position, instead of the alkoxyamino group found in compounds **1**. This generally results in reduced cytotoxicity and improved selectivity for antiprotozoal activity. Agelasine analog **2a** is a quite selective hit with respect to inhibition of *T. cruzi*. Since only three compounds of this class (**2a** – **2c**) were included in this initial screening, few other conclusions regarding SAR can be drawn at this point.

**Table 1:** Activity of compounds **1** – **9** against *P. falciparum, L. infantum, T. cruzi* and *T. b. brucei*, as well as MRC-5 fibroblast cells.

Compound No. *^a^*	*P. falciparum*		*L. infantum*		*T. cruzi*		*T. b. brucei*		MRC-5
	IC_50_μg/mL*^b^*(<0.2 μg/mL)*^c^*	SI*^d^*(>20)*^c^*	IC_50_μg/mL*^e^*(<0.5 μg/mL)*^c^*	SI*^d^*(>20)*^c^*	IC_50_μg/m*^f^*(<1.0 μg/mL)*^c^*	SI*^d^*(>50)*^c^*	IC_50 _μg/mL*^g^*(<0.2 μg/mL)*^c^*	SI*^d^*(>100)*^c^*	IC_50 _μg/mL
**1a**	0.46	1.9	7.5	<1	<0.079	>11	1.9	<1	0.86
**1b**	0.63	<1	0.77	<1	<0.096	>3.6	0.20	1.8	0.35
**1c**	0.74	<1	0.99	<1	<0.11	>2.8	<0.11	>2.8	0.31
**1d**	0.10	20	0.093	22	0.11	18	0.23	8.7	2.0
**1e**	4.2	<1	5.4	<1	1.4	2.3	0.29	11	3.2
**1f**	0.26	1.0	0.27	<1	<0.12	>2.2	<0.12	>2.2	0.26
**1g**	5.3	<1	12	<1	0.81	<1	1.0	<1	0.49
**1h**	10	<1	18	<1	3.0	1.2	1.7	2.1	3.5
**1i**	3.5	<1	1.2	<1	1.3	<1	0.29	3.2	0.92
**1j**	0.30	<1	0.23	<1	<0.11	>1.9	0.11	1.9	0.21
**1k**	7.3	<1	1.3	<1	<0.13	>2.2	<0.13	>2.2	0.28
**1l**	1.3	<1	12	<1	0.14	3.8	<0.13	>4.1	0.53
**1m**	0.69	<1	0.99	<1	<0.12	>4	<0.12	>4	0.48
**1n**	0.29	16	0.63	7.1	0.49	9.2	0.30	15	4.5
**1o**	0.94	<1	4.0	<1	<0.12	>3.8	<0.12	>3.8	0.45
**2a**	2.9	>9.0	>26	^_^	0.43	>60	13	>2	>26
**2b**	0.96	<1	2.9	<1	<0.12	>6.3	0.23	3.3	0.75
**2c (Agelasine D)**	0.29	23	1.5	4.5	4.5	1.5	0.90	7.4	6.7
**3a**	10	1.1	>26	<1	2.5	4.4	2.3	4.8	11
**3b**	3.9	3.1	>29	<1	3.6	3.3	2.6	4.6	12
**3c**	1.8	<1	2.7	<1	<0.13	>2.4	<0.13	>2.4	0.31
**3d**	2.3	1.1	>36	<1	2.2	1.1	1.2	2.1	2.5
**3e**	3.5	6	>44	<1	4.5	3.3	1.3	12	15
**3f**	>29	<1	>29	<1	0.77	3.5	2.7	1.0	2.7
**3g**	>26	^_^	9.8	>2.7	0.97	>27	2.9	>9.0	>26
**4a**	9.4	<1	>26	<1	2.3	1.1	3.5	<1	2.6
**4b**	>28	<1	>28	<1	0.28	7.5	0.89	2.4	2.1
**4c**	25	<1	>33	<1	1.5	1.0	3.8	<1	1.5
**5**	0.97	7.8	<0.11	>69	<0.11	>69	<0.11	>69	7.6
**6**	1.0	2.0	2.5	>69	<0.12	>17	0.19	11	2.0
**7**	10	1.6	>32	>69	11	1.5	4.1	3.9	16
**8**	3.37	>69	13	>69	0.19	12	0.63	3.7	2.3
**9**	>28	^_^	>28	^_^	3.2	>8.8	14	>2	>28

(a) The structures can be found in Figure 1; (b) Chloroquine 0.04 μg/mL; (c) Activity/safety criteria for an antiprotozoal hit according to TDR [28]; (d) SI = IC_50_(fibroblast)/IC_50_(parasite); (e) Miltefosine 0.24 μg/mL; (f) Benznidazole 0.25 μg/mL; (g) Melarsoprol 0.005 μg/mL.

Compounds **3a** – **3e** are neutral isomers of compounds **1**. In general, they exhibit rather low antiprotozoal activities, with exception of compound **3c**, which is a potent but non-selective inhibitor of *Trypanosoma* sp. 9-Geranylgeranyladenine **3f** was a quite selective *T. cruzi* inhibitor with only moderate toxicity.

*N*-9 Dealkylation of compounds **1** and **2** results in derivatives with the general structure **4**. This modification was particularly detrimental for antiplasmodial and antileishmania activity, while antitrypanosomal activities were also somewhat reduced and toxicity towards MRC-5 cells was virtually unchanged or even increased (cf. **4c** with **1e**).

The more interesting compound among the miscellaneous structures **5** – **9** is the imidazole derivative **5**. Compared to the agelasine analogs **1d** – **1f**, the whole pyrimidine ring is removed. This modification results in a less toxic compound with a broad antiprotozoal spectrum*.* It is worth noting that the benzimidazole derivative **6** is more toxic (MRC-5) and less active against *L. infantum* compared to the corresponding imidazole **5**.

## Conclusions

Agelasine D and several agelasine analogs and related structures were screened for inhibitory activity against *P. falciparum, L. infantum, T. b. brucei* and *T. cruzi*, as well as for cytotoxicity on MRC-5 fibroblast cells. Many compounds displayed high general toxicity. Nevertheless, two compounds (**1d** and **5**) were identified as hits for leishmaniasis and two (**2a** and **5**) for Chagas disease*.* Identification of the hits as well as other SAR data from this initial screening will be valuable for design of more potent and selective potential drugs against these neglected tropical diseases.

## Experimental

### Compounds

All compounds studied were synthesized as described before; **1a**, **1d**, and **3b** [20], **1b**-**1c**, **1e**-**1k**, **2a**-**2b**, **3a**, **3c**-**3g**, **4**-**8** [23], **1l**-**1m**, **9** [22], **1n**-**1o**, **2c** [19]. Stock solutions were prepared in 100% DMSO at 20 mg/mL.

### Test plate production

The experiments were performed in 96-well plates (Greiner) at four-fold dilutions in a dose-titration range of 64 μg/mL to 0.25 μg/mL. Dilutions were carried out by a programmable precision robotic station (BIOMEK 2000, Beckman, USA). Each plate also contained medium-controls (blanks: 0% growth), infected untreated controls (negative control: 100% growth) and reference controls (positive control). Tests were run in duplicate in two independent experiments.

### Biological screening tests

The integrated panel of microbial screens for the present study and the standard screening methodologies were adopted as have been described before [29]. Compounds with high cytotoxicity and related aspecificity across the different protozoa models were not titrated down to the exact IC_50_.

### Antiplasmodial activity

The chloroquine-suceptible *P. falciparum* GHA-strain was used. Parasites were cultured in human erythrocytes A^+^ at 37 °C under a low oxygen atmosphere (3% O_2_, 4% CO_2_, and 93% N_2_) in a modular incubation chamber [30]. The culture medium was RPMI-1640, supplemented with 10% human serum. Two hundred microliters of infected human red blood cells suspension (1% parasitemia, 2% hematocrit) were added to each well of the plates with test compounds and incubated for 72 h. After incubation, test plates were frozen at -20 °C. Parasite multiplication was measured by the Malstat method [31]. One hundred microliters of Malstat reagent were transferred in a new plate and mixed with 20 μL of the hemolysed parasite suspension for 15 minutes at room temperature. After addition of 20 μL NBT/PES solution and 2 h incubation in the dark, the absorbance was spectrophotometrically read at 655 nm (Biorad 3550-UV microplate reader). Percentage growth inhibition was calculated compared to the negative blanks.

### Antitrypanosomal activity

*T. b. brucei:* Trypomastigotes of *T. b. brucei* Squib-427 strain (suramin-sensitive) were cultured at 37 °C and 5% CO_2_ in Hirumi-9 medium [32], supplemented with 10% fetal calf serum (FCS). Assays were performed by adding 1.5×10^4^ trypomastigotes/well. After 72 h incubation, parasite growth was assessed fluorimetrically by adding resazurin [33] for 24 h at 37 °C. Fluorescence was measured using a GENios Tecan fluorimeter (excitation 530 nm, emission 590 nm).

*T. cruzi*: Tulahuen CL2 strain (nifurtimox-sensitive) was maintained on MRC-5 cells in minimal essential medium (MEM) supplemented with 20 mM L-Glutamine, 16.5 mM sodium hydrogen carbonate and FCS (5%) at 37 °C and 5% CO_2_. To determine *in vitro* anti-trypanosomal activity, 4×10^3^ MRC-5 cells and 4×10^4^ parasites were added to each well of test plate with compound. After incubation at 37 °C for 7 days, parasite growth was assessed by adding of β-galactosidase substrate, chlorophenol red β-D-galactopyranoside [34] for 4 h at 37 °C. The color reaction was read at 540 nm and absorbance values were expressed as a percentage of the blank controls.

### Antileishmanial activity

*Leishmania infantum* amastigotes (MHOM/ET 67) were collected from an infected donor hamster and used to infect primary peritoneal mouse macrophages. To determine *in vitro* antileishmanial activity, 3×10^4^ macrophages were seeded in each well of a 96-well plate. After 48 h outgrowth, 5×10^4^ amastigotes/well were added and incubated for 2 h at 37 °C. Pre-diluted compounds were subsequently added and the plates were further incubated for 120 h at 37 °C and 5% CO_2_. Parasite burdens were determined microscopically after Giemsa staining and expressed as a percentage of the blank controls without compound.

### Cytotoxicity assay

MRC-5 SV_2_ cells, human fetal lung fibroblast, were cultivated in MEM, supplemented with L-glutamine (20 mM), 16.5 mM sodium hydrogen carbonate and 5% FCS at 37 °C and 5% CO_2_. For the assay, 10^4^ MRC-5 cells/well were seeded onto the test plates containing the pre-diluted compounds and incubated at 37 °C and 5% CO_2_ for 72 h. Cell viability was determined after addition of resazurin.

## References

[1] Gelb M.H. (2007). Drug discovery for malaria: a very challenging and timely endeavor. Curr. Opin. Chem. Biol..

[2] Tuteja R. (2007). Malaria – an overview. FEBS J..

[3] Croft S.L., Barrett M.P., Urbina J.A. (2005). Chemotherapy of trypanosomiases and leishmaniasis. Trends Parasitol..

[4] Chappuis F., Sundar S., Hailu A., Ghalib H., Rijal S., Peeling R.W., Alvar J., Boerlaert M. (2007). Visceral leishmaniasis: what are the needs for diagnosis, treatment and control?. Nature. Rev. Microbiol..

[5] Barrett M.P., Boykin D.W., Brun R., Tidwell R.R. (2007). Human African trypanosomiasis: pharmacological re-engagement with a neglected disease. J. Pharmacol..

[6] Teixeira A.R.L., Nitz N., Giumaro M.C., Gomes C., Santos-Buch C.A. (2006). Chagas disease. Postgrad. Med. J..

[7] Nwaka S., Hudson A. (2006). Innovative lead discovery strategies for tropical diseases. Nature Rev. Drug. Discov..

[8] Plubrukarn A. (2006). Marine invertebrates: a diverted approach towards the treatment of tropical diseases. Recent. Prog. Med. Plants.

[9] Capon R.J., Faulkner D.J. (1984). Antimicrobial metabolites from a Pacific sponge, *Agelas* sp.. J. Am. Chem. Soc..

[10] Nakamura H., Wu H., Ohizumi Y., Hirata Y. (1984). Agelasine-A –B, -C and –D, novel bicyclic diterpenoids with a 9-methyladeninium unit possessing inhibitory effects on Na,K-ATPase isolated from the Okinawian sea sponge *Agelas* sp.. Tetrahedron Lett..

[11] Wu H., Nakamura H., Kobayashi J., Ohizumi Y. (1984). Agelasine-E and –F, novel monocyclic diterpenoids with a 9-methyladeninium unit possessing inhibitory effects on Na,K-ATPase isolated from the Okinawian sea sponge *Agelas nakamurai* Hoshino. Tetrahedron Lett..

[12] Wu H., Nakamura H., Kobayashi J., Kobayashi M., Ohizumi Y., Hirata Y. (1986). Structures of agelasines, diterpenes having a 9-methyladeninium chromophore isolated from the Okinavian marine sponge *Agelas nakamurai* Hoshino. Bull. Chem. Soc. Jpn..

[13] Ishida K., Ishibashi M., Shigemori H., Sasaki T., Kobayashi J. (1992). Agelasine G, a new antileukemic alkaloid from the Okinavian marina sponge *Agelas* sp.. Chem. Pharm. Bull..

[14] Hattori T., Adachi K., Shizuri Y. (1997). New agelasine compound from the marine sponge *Agelas mauretania* as an antifouling substance against macroalgae. J. Nat. Prod..

[15] Fu X., Schmitz F.J., Tanner R.S., Kelly-Borges M. (1998). Agelasines H and I, 9-methyladenine-containing diterpenoids from *Agelas* sponge. J. Nat. Prod..

[16] Iwagawa T., Kaneko M., Okamura H., Nakatani M., van Soest R.W.M. (1998). New alkaloids from the Papua New Guinean sponge *Agelas nakamurai*. J. Nat. Prod..

[17] Appenzeller J., Michi G., Martin M.-T., Gallard J.-F., Menou J.-L., Boury-Esnault N., Hooper J., Petek S., Chevalley S., Valentin A., Zaparucha A., Al-Mourabit A., Debitus C. (2008). Agelasines J, K, and L from the Solomon Islands marine sponge *Agelas* cf. *mauritiana*. J. Nat. Prod..

[18] Utenova B.T., Gundersen L.-L. (2004). Synthesis of (+)-agelasine D from (+)-manool. Tetrahedron Lett..

[19] Vik A., Hedner E., Charnock C., Samuelsen Ø., Larsson R., Gundersen L.-L., Bohlin. L (2006). (+)-Agelasine D: improved synthesis and evaluation of antibacterial and cytotoxic activities. J. Nat. Prod..

[20] Bakkestuen A.K., Gundersen L.-L., Petersen D., Utenova B.T., Vik A. (2005). Synthesis and antimycobacterial activity of agelasine E and analogs. Org. Biol. Chem..

[21] Proszenyák Á, Brændvang M., Charnock C., Gundersen L.-L. (2009). The first synthesis of *ent*-Agelasine F. Tetrahedron.

[22] Proszenyák Á, Charnock C., Hedner E., Larsson R., Bohlin L., Gundersen L.-L. (2007). Synthesis and antimicrobial and antineoplastic activities of agelasine and agelasimine analogs with a β-cyclocitral derived substituent. Arch. Pharm. Chem. Life Sci..

[23] Vik A., Hedner E., Charnock C., Tangen L.W., Samuelsen Ø., Larsson R., Bohlin. L., Gundersen L.-L. (2007). Antimicrobial and cytotoxic activity of agelasine and agelasimine analogs. Bioorg. Med. Chem..

[24] Roggen H., Gundersen L.-L. (2008). Synthetic studies directed towards agelasine analogs. Synthesis, tautomerism, and alkylation of 2-substituted *N*-methoxy-9-methyl-9*H*-purin-6-amines. Eur. J. Org. Chem..

[25] Sjögren M., Dahlström M., Hedner E., Jonsson P.R., Vik A., Gundersen L.-L., Bohlin L. (2008). Antifouling activity of the sponge metabolite agelasine D and synthesized analogs on *Balanus improvisus*. Biofouling.

[26] Fathi-Afshar R., Allen T.M. (1988). Biological active metabolites from *Agelas mauretania*. Can. J. Chem..

[27] Fathi-Afshar R., Allen T.M. (1989). Some pharmacological activities of novel adenine-related compounds isolated from a marine sponge *Agelas mauretania*. Can. J. Physiol. Pharmacol..

[28] (2007). TDR, Lead discovery for drugs.

[29] Cos P., Vlietinck A.J., Berghe D.V., Maes L. (2006). Anti-infective potential of natural products: How to develop a stronger *in vitro* ‘proof-of-concept’. J. Ethnopharmacol..

[30] Trager W., Jensen J.B. (1976). Human malaria parasites in continuous culture. Science.

[31] Makler M.T., Ries J.M., Williams J.A., Bancroft J.E., Piper R.C., Gibbins B.L., Hinrichs D.J. (1993). Parasite lactate dehydrogenase as an assay for *Plasmodium falciparum* drug sensitivity. Am. J. Trop. Med. Hyg..

[32] Hirumi H., Hirumi K. (1989). Continuous cultivation of *Trypanosoma brucei* blood stream forms in a medium containing a low concentration of serum protein without feeder cell layers. J. Parasitol..

[33] Raz B., Iten M., Grether-Buhler Y., Kaminsky R., Brun R. (1997). The Alamar Blue asssay to determine drug sensitivity of African trypanosomes (*T. b. rhodesiense*, *T. b. gambiense*) *in vitro*. Acta Trop..

[34] Buckner F.S., Verlinde C.L., La Flamme A.C., Van Voorhis W.C. (1996). Efficient technique for screening drugs for activity against *Trypanosoma cruzi* using parasites expressing beta-galactosidase. Antimicrob. Agents Chemother..

